# Use of surgical bone cement to increase the projection of the nasomaxillary buttress in a case of orthognathic surgery. Technical note

**DOI:** 10.4317/jced.60446

**Published:** 2023-05-01

**Authors:** Íñigo Aragón-Niño, Jorge Guiñales-Diaz de Cevallos, José-Luis Del Castillo-Pardo de Vera, Alba García-López-Chicharro, Carolina Cuesta-Urquía, Marta-María Pampín-Martínez, José-Luis Cebrián-Carretero

**Affiliations:** 1 Oral and Maxillofacial Surgery Department. La Paz University Hospital. Madrid, Spain

## Abstract

The soft tissue outcome of the projection at the level of the nasomaxillary buttress is difficult to manage in cases of severe hypoprojection, being orthognathic surgery resolutive at the occlusal level but sometimes insufficient at the level of esthetic outcome. The literature describes the use of alloplastic prostheses and autologous bone grafts, but there are few documented cases of the use of premolded surgical cement for this purpose. The main advantage of the use of bone cement over the alternatives described is its ability to be premolded for customization, low cost, easy availability, speed of preparation and minimal comorbidity. This technical note describes the surgical steps and outcome of the use of surgical bone cement for projection augmentation at this level, including notes on preparation, premolding and fixation.

** Key words:**Orthognathic surgery, maxillary surgery, surgical bone cement, nasomaxillary buttress.

## Introduction

Current orthognathic surgery enables precise control of the expected outcome at the occlusal and osseous levels. Advances in digital diagnosis and virtual surgical planning have elevated the technique to a high level of precision. However, a significant concern is the impact of these changes on soft tissues, where virtual planning is less precise (1).

This can result in lower soft tissue projection than expected in certain anatomical areas, leading to suboptimal aesthetic outcomes. This is particularly apparent in the nasomaxillary buttress, where the complex intersection of anatomical structures makes it challenging to manipulate without affecting other critical structures, such as the insertion of the nasal ala, tip projection, and nasal spine position (2,3).

In these cases, a rapid solution that does not require pre-planning, provides a customized outcome adapted to the patient, and delivers immediate surgical results is needed. Additionally, cost-effectiveness and accessibility are also crucial considerations.

This technical note outlines the use of surgical bone cement to design customized prostheses and implant them in the nasomaxillary buttress to address hypoprojection in a patient undergoing bimaxillary orthognathic surgery.

## Case Report

Once the osteosynthesis is completed and correct occlusion is verified, before closing the maxillary approach, radiopaque bone cement Surgical Simplex®P from Stryker Orthopaedics (Howmedica Osteonics Corp. Mahwah, NJ, USA) is used to create two elements of bone projection at the level of the nasomaxillary buttresses.

This medical product is supplied packaged in two sterile components: an ampoule of a liquid monomer and a package containing a powder component. At the time of use the powder and liquid are mixed. This mixture is prepared for exothermic polymeric formation of a soft and malleable mass which after a few minutes forms a hard structure.

The standard indication for surgical bone cement is the fixation of prostheses in bone in orthopedic musculoskeletal surgeries and also for the fixation of pathological fractures with loss of substance. The indication described in this technical note is an off-label use.

**Figure 1:**
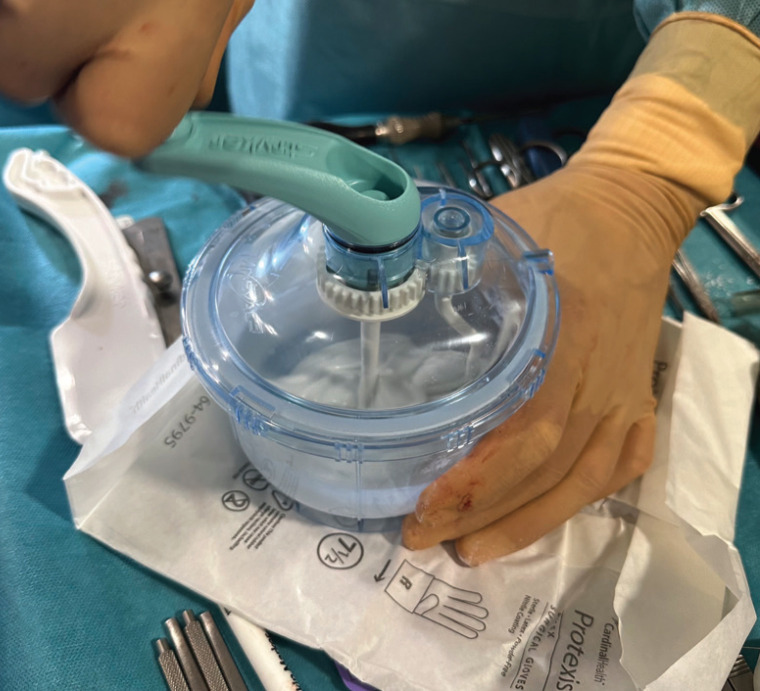
Mixture of elements for the creation of the surgical bone cement.

During the preparation process, the contents of the powder package are emptied into a sterile mixing device and the contents of the ampoule containing the liquid component are added. The mixture is then stirred using the device until the powder is completely mixed with the liquid and a non-sticky mass that does not adhere to the surgical gloves of the operator is obtained. This process takes approximately 5 minutes, (Fig. 1).

**Figure 2:**
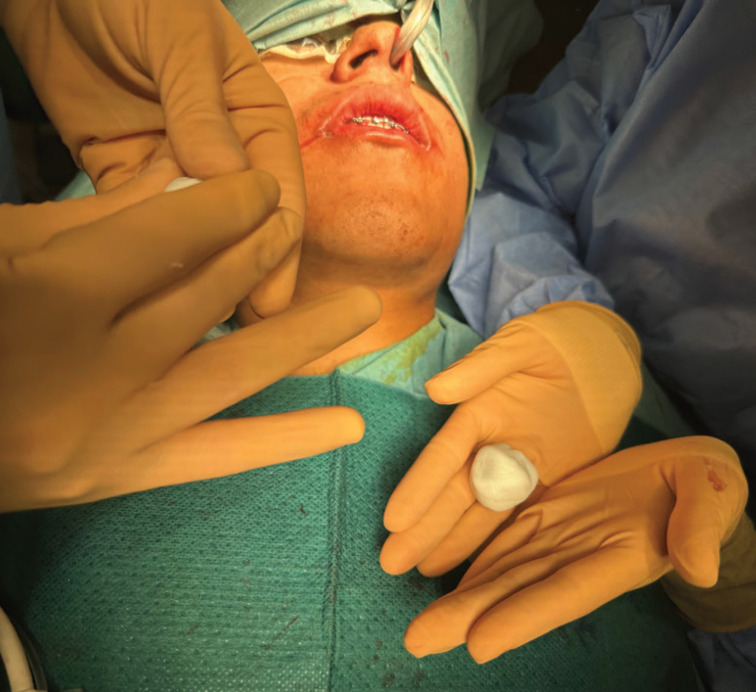
Manual premolding of the cement.

After obtaining the mass, the molding process begins, taking a small part of the product and giving it the desired shape, in this case an irregular oval shape with a central projection, (Fig. 2).

Once preformed, the structures are placed on the patient’s maxillary bone and the soft tissue projection is determined. At this point it is useful to apply the product on one side only and compare the difference obtained against the contralateral side.

**Figure 3:**
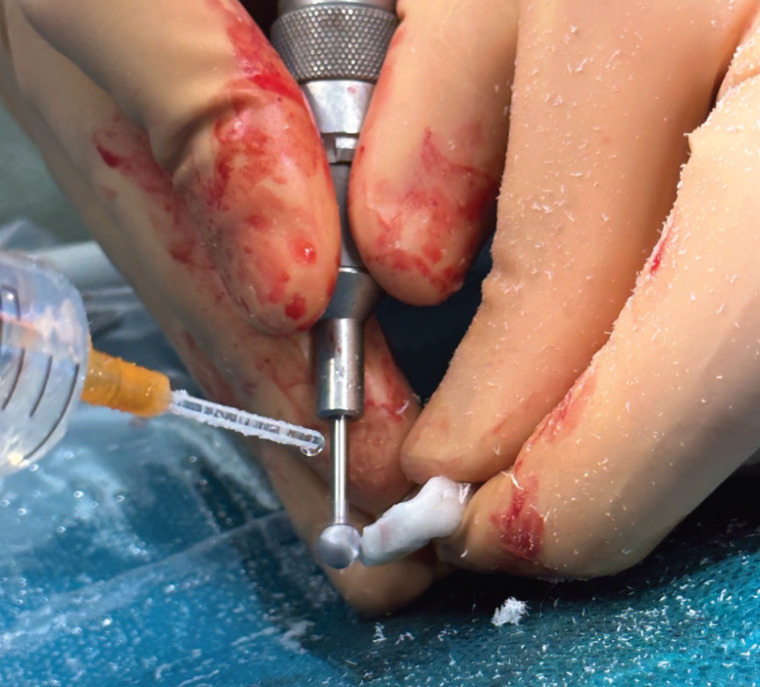
Remodeling with handpiece and turbine.

Then, the necessary pre-molding adjustments are made until the desired result is obtained. After applying the product, the position of the element must be kept fixed without movements until the cement sets. Once this occurs, final small modifications can be made in shape with a handpiece and milling turbine, (Fig. 3).

**Figure 4:**
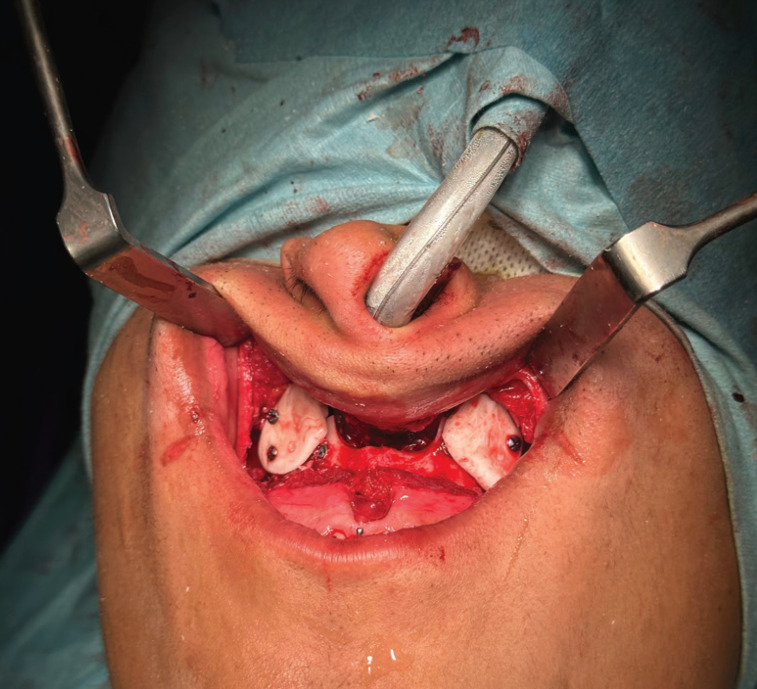
Final result fixed with osteosynthesis screws.

Once the two projection elements have been obtained and after verifying that they produce the desired effect on the soft tissues, they are fixed to the maxillary bone by using osteosynthesis screws to ensure long-term stability, (Fig. 4).

Finally, the maxillary approach is closed and the surgery is finished. As postoperative care in addition to the orthognathic surgery itself, it is only necessary to extend the postoperative prophylactic antibiotherapy for up to 10 days.

## Discussion

Orthognathic surgery is the gold standard for the correction of dentofacial deformities with a good correlation between pre-surgical planning and the results obtained. Sometimes, in spite of obtaining a good result at occlusal level and a correction of the deformity, there are still some deficits of projection at soft tissue level that results in a suboptimal aesthetic outcome (4,5).

The correction of these projection deficits is usually performed with alloplastic materials (6) or with autologous bone graft (7,8). The use of alloplastic materials has as its main disadvantage the difficult adaptation to the patient’s bone contour, especially if this has been modified by the intervention itself, and its high cost. The use of bone grafting has as disadvantages the comorbidity and the difficult handling for premolding.

The use of surgical bone cement is an alternative for this purpose that overcomes all the mentioned disadvantages because it allows complete adaptability, unlimited possibilities of premolding and customization to the patient and the case and minimal comorbidity. Taking into account its reduced cost, we can consider it a recommendable and cost-effective option to increase soft tissue projection at the level of the nasomaxillary buttresses in selected cases undergoing orthognathic surgery.
